# Supplementary material for: Variation in detected adverse events using trigger tools: A systematic review and meta-analysis
Source: PLoS One. 2022 Sep 1;17(9):e0273800. doi: 10.1371/journal.pone.0273800 (PMC9436152; doi:10.1371/journal.pone.0273800)
Supplement: S1 File — (PDF) [file pone.0273800.s002.pdf]

Quality Assessment Tool for General Trigger Tool studies

State your review question here, and answer all signaling questions and judgments in light of this review question.

Aim: To explore variations in adverse events detection rates using the trigger tool methodology in acute care settings.

P patient records to patients hospitalized at least 24 hours (inpatient) with any specific disease admitted to any ward.

I Global Trigger Tool or a modified version (added/removed/modified triggers)

C not applicable

O prevalence overall, by type of AE, and by type of hospital or ward.

DOMAIN 1A: PATIENT SELECTION

Describe methods of patient selection:

A. Risk of bias*: Could the selection of patient records have introduced bias?*

1. ***Was a consecutive or random sample of patient records enrolled?***
   Yes / No / Unclear
   Reflect if all the subjects selected or recruited were from the same or similar populations (including the same time period)? Were inclusion and exclusion criteria for being in the study prespecified and applied uniformly to all participants in a consecutive manner? If all accessible patient records were selected as sample or if the process of sampling was done with the method of random sampling, the question will be answered as “yes”.

2. ***Did the study consider patients covering a broad range of indications for hospitalizations? Yes / No / Unclear***
   This question will be answered with “no” when patients with very different profiles are not considered either by exclusion at study entry or by exclusion from statistical analysis. Such exclusions are highly likely to alter the estimates of prevalence. This is a situation, where GTT might over/underestimates adverse events. For example: exclusion of certain group of patients due to a long length of stay or with lots of transfers.

RISK: LOW / HIGH / UNCLEAR

B. Applicability*: Are there concerns that the included patients and setting do not match the review question?*

If a study did not meet the patient population or setting as described in the review question, there will be a high concern regarding its applicability. In this specific review, we allow for a broad range of settings and study populations.

CONCERN: LOW / HIGH / UNCLEAR

DOMAIN 1B: RATER/REVIEWER

Describe methods to sample patients to be included in the reliability estimations:<br><br>*State the determined number of reviewers:*<br><br>*State the number of patient records included in the reliability estimations:*<br><br>*State the number of replicate observations:*<br><br>*Describe the reviewer characteristics (e.g., training, experience):*<br><br>*Did raters judge/analyze the records independently (e.g., stage 1 screening)?*

***A. Risk of bias**: Could the selection of raters have introduced bias?*

1. ***Were reviewer(s) selected based on his/her experience and/or professional background in the clinical setting? Yes / No / Unclear***
   The lack of experience of the reviewer(s) in the clinical setting may introduce bias. For reviewers with appropriate clinical background the bias might be lower.

2. ***Were reviewer(s) trained on using trigger tool methodology and application? Yes / No / Unclear***
   The lack of GTT training in applying the GTT may introduce bias. For reviewer with more training the bias might be lower.

3. **Do reviewers have experience in applying the GTT or another retrospective chart review methodology? *Yes / No / Unclear***
   The lack of GTT experience in applying the GTT may introduce bias. For reviewer with more GTT experience the bias might be lower.

4. **Were reliability/agreement assessments conducted during training for the trigger tool application? *Yes / No / Unclear***
   If reliability was not assessed during the training phase, bias might have been introduced for the AE detection.

RISK: LOW / HIGH / UNCLEAR

***B. Applicability**: Are there concerns that the raters do not match the review question?*

For example, if the profile of raters applying GTT in the study substantially differ from the profile of health care professionals that would apply GTT in clinical practice, a high concern may arise.

CONCERN: LOW / HIGH / UNCLEAR

DOMAIN 2: TRIGGER TOOL METHOD

Description of the method:

A. Risk of Bias*: Could the conduct or interpretation of the trigger tool have introduced bias?*

1. **Was the modification of triggers/selection of new triggers based on literature review and/or by a group of experts in the field?** ***Yes / No / Unclear***
Empirical evidence or at least a biomedical rationale should exist for each of the triggers included in the tool. This particularly applies for *new triggers not part of the original IHI set of triggers.*

2. **Are the triggers used the same for all settings and/or the hospitals participating in the study?** ***Yes / No / Unclear***
If not the same across participating units, a strong rational should have been given to adapt the triggers. For example, it is acceptable to adapt triggers according to national circumstances where a specific drug is not licensed.

3. **Was the presence of any adverse event checked/controlled at the admission of the patient to the unit/hospital?** ***Yes / No / Unclear***
If not, the unit/hospital might think that those AEs were the consequences of their own care to the patients.

RISK: LOW / HIGH / UNCLEAR

B. Applicability*: Are there concerns that the trigger tool, its conduct, or its interpretation differ from the review question?*

If test conduct, technology, setting, or interpretation differ from your review question the results may not be applicable. For example: triggers are not related to the IHI GTT; AEs captured in the study are more a trigger than an AE.

CONCERN: LOW / HIGH / UNCLEAR

DOMAIN 3: OUTCOMES

***A. Risk of Bias**: Could the definition of outcomes have introduced bias?*

Description the definition of AE:<br><br>*Describe the definition of harm:*<br><br>*Describe how prevalence was measured:*<br><br>*Describe your judgment, if you deem the definitions to be standard, or deviating from our review definitions and describe if you deem reliability sufficiently high:*

1. ***Were the adverse events defined based on the U.S Food & Drug Administration (FDA) definition and/or on the Institute for Healthcare Improvement (IHI) definition? Yes / No / Unclear***
   - FDA definition: "Adverse event means any untoward medical occurrence associated with the medical intervention(s) at the hospital, whether or not considered drug related." (https://www.accessdata.fda.gov/scripts/cdrh/cfdocs/cfcfr/CFRSearch.cfm?fr=312.32)
   - IHI definition: "unintended physical injury resulting from or contributed by medical care that requires additional monitoring, treatment or hospitalization, or that results in death." (Griffin FA, Resar RK. IHI Global Trigger Tool for measuring adverse events. Institute for Healthcare Improvement Innovation Series White Paper. 2009.)

2. ***Was the assessment of severity of the adverse events based on the National Coordinating Council for Medication Error Reporting and Prevention (NCC MERP)? Yes / No / Unclear***
   For classification of the severity of the AEs follow:
   http://www.nccmerp.org/sites/default/files/indexColor2001-06-12.pdf

3. ***For prevalence, were the outcome measures (dependent variables) clearly defined? Yes / No / Unclear***

4. ***For prevalence, were definitions consistently applied across all study participants? Yes / No / Unclear***

RISK: LOW / HIGH / UNCLEAR

DOMAIN 4: FLOW AND TIMING

***A. Risk of Bias**: Could the patient flow have introduced bias?*

1. **Was the completeness of health records data discussed and addressed?** ***Yes / No / Unclear***
   If yes, the risk of bias will be lower since it takes into account missing data for the analysis.

2. **Were all patients included in the analysis of prevalence? *Yes / No / Unclear***
   This question will be scored as "yes" if all patients who were recruited into the study were included in the analysis. No is scored if one or more patients are missing. As the study prevalence of AEs can be as low as 2.x%, we judge high risk of bias if 6% or more of the patients are missing, assuming that one third of the missing would not be at random, potentially affecting our estimates of the prevalence.

RISK: LOW / HIGH / UNCLEAR
