# Supplementary material for: Variation in detected adverse events using trigger tools: A systematic review and meta-analysis
Source: PLoS One. 2022 Sep 1;17(9):e0273800. doi: 10.1371/journal.pone.0273800 (PMC9436152; doi:10.1371/journal.pone.0273800)
Supplement: S1 Table — (PDF) [file pone.0273800.s003.pdf]

S1 Table. Assessments of risk of bias and applicability-related concerns (sorted alphabetically)

Study	Risk of bias					Applicability-related concerns		
	Patient selection	Reviewer	Trigger tool method	Outcome	Flow and timing	Patient selection	Reviewer	Trigger tool method
Asavaroengchai 2009 [1]	Low	Unclear	Unclear	Low	Low	High	Unclear	Low
Bjorn 2017 [2]	Low	Low	Low	Low	Low	Low	Low	Low
Brösterhaus 2020 [3]	Low	Low	Low	Low	High	Low	Low	Low
Cihangir 2013 [4]	Low	Low	Unclear	Low	Low	Low	Low	Unclear
Classen 2011 [5]	Low	Unclear	Low	Low	Low	Low	Low	Low
Croft 2016 [6, 7]	High	Unclear	Low	Low	Low	High	High	Low
Deilkas 2015 [8-10]	Low	Unclear	Unclear	Low	Low	Low	Low	Low
Deilkas 2017 [11]	Low	Low	Low	Low	Low	Low	Low	Low
Farup 2015 [12]	Low	Unclear	Unclear	Low	Unclear	Low	Unclear	Low
Garrett 2013 [13, 14]	Low	Low	Unclear	Low	Low	Low	Low	Low
Gerber 2020 [15]	High	Unclear	Low	Low	Low	Low	Low	Low
Griffin 2008 [16]	Low	Unclear	Low	Low	Low	Low	Low	Low
Grossmann 2019 [17]	Low	Low	Low	Low	Low	Low	Low	Low
Guzman Ruiz 2015 [18, 19]	Low	High	Unclear	Low	Low	Low	Unclear	Unclear
Haukland 2017 [20, 21]	Low	Unclear	Unclear	Low	Low	Low	Low	Low
Hoffmann 2018 [22]	Low	Low	Low	Low	Low	High	Low	Low
Hommel 2020 [23-25]	Unclear	Low	Low	Low	Low	Low	Low	Low
Hu 2019 [26]	Low	Unclear	Unclear	Low	Low	Low	High	Low
Hwang 2014 [27]	Low	High	Unclear	Low	Low	Low	Unclear	Low
Kaibel 2020 [28]	High	Unclear	Low	Low	Unclear	Low	High	Low
Kelly-Pettersson 2020 [29]	High	Low	Low	Low	Low	Low	Unclear	Low
Kennerly 2014 [30-32]	High	Unclear	Low	Low	Unclear	Low	Unclear	Low
Kurutkan 2015 [33]	Low	Unclear	Low	Low	Low	High	Unclear	Low
Landrigan 2010 [34, 35]	Low	Low	Low	Low	Low	Low	Low	Low
Lipczak 2011 [36, 37]	Low	Unclear	Low	High	Unclear	High	Unclear	Low
Lipitz-Snyderman 2017 [38]	Low	Unclear	Unclear	Low	Low	Low	Unclear	Low
Mattson 2014 [39, 40]	Low	Low	Low	Low	Low	Low	Low	Low
Mayor 2017 [41]	Low	Unclear	Unclear	Low	Unclear	Low	Unclear	Unclear
Menendez-Fraga 2021 [42]	Low	Low	Low	Low	Unclear	Low	Low	Low
Mevik 2016 [43, 44]	Low	Low	Low	Low	Low	Low	Low	Low
Moraes 2021 [45]	Low	Low	Low	Low	Low	Low	Low	Low
Mortaro 2017 [46]	Low	Low	Low	Low	Low	Low	Low	Low

Study	Risk of bias					Applicability-related concerns		
	Patient selection	Reviewer	Trigger tool method	Outcome	Flow and timing	Patient selection	Reviewer	Trigger tool method
Mull 2015 [47]	Low	Unclear	Low	Low	Low	Low	Unclear	Low
Müller 2016 [48]	High	High	Low	Low	Unclear	Low	High	Low
Naessens 2010 [49, 50]	Low	Unclear	Unclear	Low	Low	Low	Low	Low
Najjar 2013 [51]	Low	Low	Low	Low	Low	Low	Low	Low
Nilsson 2016 [52]	Low	Low	High	Low	Unclear	Low	Low	Low
Nilsson 2018 [53, 54]	Low	Low	Low	Low	Low	Low	Low	Low
Nowak 2022 [55]	High	Low	Low	Low	Low	High	Low	Low
O'Leary 2013 [56]	Low	Low	Low	High	Low	Low	Low	Low
Perez Zapata 2015 [57, 58]	Low	High	Low	Low	Low	Low	Low	Low
Perez Zapata 2022 [59]	Unclear	High	Low	Low	Low	Low	Unclear	High
Pierdevara 2020 [60]	Low	High	Low	Low	Low	Low	Unclear	Low
Rutberg 2014 [61]	Low	Unclear	Low	Low	Low	Low	Low	Low
Rutberg 2016 [62]	Low	Low	Low	Low	Low	High	Low	Low
Schildmeijer 2012 [63]	Low	Low	Low	Unclear	Low	High	Low	Low
Sekijima 2020 [64]	High	Unclear	Low	Low	Low	Low	Unclear	Low
Suarez 2014 [65, 66]	Low	Unclear	Low	Low	Low	Low	Low	Low
Toribio-Vicente 2018 [67]	Low	High	Unclear	Low	Low	Low	Low	Unclear
Unbeck 2013 [68]	Low	Low	Unclear	Low	Low	Low	Low	Low
von Plessen 2012 [69]	Low	Low	Low	Low	Unclear	Low	Low	Low
Wilson 2012 [70]*	Unclear	Low	Unclear	High	High	High	Low	Unclear
Xu 2020 [71]	Low	Low	Low	Low	Low	Low	Low	Low
Zadvinskis 2018 [72]	Low	Unclear	Unclear	Low	Low	Low	Unclear	Unclear

References

1. Asavaroengchai S, Sriratanaban J, Hiransuthikul N, Supachutikul A. Identifying adverse events in hospitalized patients using global trigger tool in Thailand. Asian Biomedicine. 2009;3(5):545-50.
2. Bjorn B, Anhoj J, Ostergaard M, Kodal AM, von Plessen C. Test-Retest Reliability of an Experienced Global Trigger Tool Review Team. J Patient Saf. 2017. Epub 2017/10/13. doi: 10.1097/PTS.0000000000000433.
3. Brosterhaus M, Hammer A, Kalina S, Grau S, Roeth AA, Ashmawy H, et al. Applying the Global Trigger Tool in German Hospitals: A Pilot in Surgery and Neurosurgery. J Patient Saf. 2020;16(4):e340-e51. Epub 2020/11/21. doi: 10.1097/PTS.0000000000000576.
4. Cihangir S, Borghans I, Hekkert K, Muller H, Westert G, Kool RB. A pilot study on record reviewing with a priori patient selection. BMJ Open. 2013;3(7). Epub 2013/07/23. doi: 10.1136/bmjopen-2013-003034.
5. Classen DC, Resar R, Griffin F, Federico F, Frankel T, Kimmel N, et al. 'Global trigger tool' shows that adverse events in hospitals may be ten times greater than previously measured. Health Aff (Millwood). 2011;30(4):581-9. Epub 2011/04/08. doi: 10.1377/hlthaff.2011.0190.
6. Croft LD, Liquori M, Ladd J, Day H, Pineles L, Lamos E, et al. The Effect of Contact Precautions on Frequency of Hospital Adverse Events. Infect Control Hosp Epidemiol. 2015;36(11):1268-74. Epub 2015/08/19. doi: 10.1017/ice.2015.192.
7. Croft LD, Liquori ME, Ladd J, Day HR, Pineles L, Lamos EM, et al. Frequency of Adverse Events Before, During, and After Hospital Admission. South Med J. 2016;109(10):631-5. Epub 2016/10/06. doi: 10.14423/SMJ.0000000000000536.
8. Bjertnaes O, Deilkas ET, Skudal KE, Iversen HH, Bjerkan AM. The association between patient-reported incidents in hospitals and estimated rates of patient harm. Int J Qual Health Care. 2015;27(1):26-30. Epub 2014/11/25. doi: 10.1093/intqhc/mzu087.
9. Deilkas ET, Bukholm G, Lindstrom JC, Haugen M. Monitoring adverse events in Norwegian hospitals from 2010 to 2013. BMJ Open. 2015;5(12):e008576. Epub 2016/01/01. doi: 10.1136/bmjopen-2015-008576.
10. Mevik K, Hansen TE, Deilkas EC, Ringdal AM, Vonen B. Is a modified Global Trigger Tool method using automatic trigger identification valid when measuring adverse events? Int J Qual Health Care. 2019;31(7):535-40. Epub 2018/10/09. doi: 10.1093/intqhc/mzy210.
11. Deilkas ET, Risberg MB, Haugen M, Lindstrom JC, Nylen U, Rutberg H, et al. Exploring similarities and differences in hospital adverse event rates between Norway and Sweden using Global Trigger Tool. BMJ Open. 2017;7(3):e012492. Epub 2017/03/23. doi: 10.1136/bmjopen-2016-012492.
12. Farup PG. Are measurements of patient safety culture and adverse events valid and reliable? Results from a cross sectional study. BMC Health Serv Res. 2015;15:186. Epub 2015/05/03. doi: 10.1186/s12913-015-0852-x.
13. Adler L, Yi D, Li M, McBroom B, Hauck L, Sammer C, et al. Impact of Inpatient Harms on Hospital Finances and Patient Clinical Outcomes. J Patient Saf. 2018;14(2):67-73. Epub 2015/03/25. doi: 10.1097/PTS.0000000000000171.
14. Garrett PR, Jr., Sammer C, Nelson A, Paisley KA, Jones C, Shapiro E, et al. Developing and implementing a standardized process for global trigger tool application across a large health system. Jt Comm J Qual Patient Saf. 2013;39(7):292-7. Epub 2013/07/31. doi: 10.1016/s1553-7250(13)39041-2.
15. Gerber A, Da Silva Lopes A, Szüts N, Simon M, Ribordy-Baudat V, Ebneter A, et al. Describing adverse events in Swiss hospitalized oncology patients using the Global Trigger Tool. Health Sci Rep. 2020;3(2):e160. Epub 2020/05/15. doi: 10.1002/hsr2.160.
16. Griffin FA, Classen DC. Detection of adverse events in surgical patients using the Trigger Tool approach. Qual Saf Health Care. 2008;17(4):253-8. Epub 2008/08/06. doi: 10.1136/qshc.2007.025080.
17. Grossmann N, Gratwohl F, Musy SN, Nielen NM, Simon M, Donze J. Describing adverse events in medical inpatients using the Global Trigger Tool. Swiss Med Wkly. 2019;149:w20149. Epub 2019/11/11. doi: 10.4414/smw.2019.20149.

18. Guzman-Ruiz O, Ruiz-Lopez P, Gomez-Camara A, Ramirez-Martin M. [Detection of adverse events in hospitalized adult patients by using the Global Trigger Tool method]. Rev Calid Asist. 2015;30(4):166-74. Epub 2015/05/31. doi: 10.1016/j.cali.2015.03.003.
19. Guzman Ruiz O, Perez Lazaro JJ, Ruiz Lopez P. [Performance and optimisation of a trigger tool for the detection of adverse events in hospitalised adult patients]. Gac Sanit. 2017;31(6):453-8. Epub 2017/05/27. doi: 10.1016/j.gaceta.2017.01.014.
20. Haukland EC, von Plessen C, Nieder C, Vonen B. Adverse events in hospitalised cancer patients: a comparison to a general hospital population. Acta Oncol. 2017;56(9):1218-23. Epub 2017/04/06. doi: 10.1080/0284186X.2017.1309063.
21. Haukland EC, Mevik K, von Plessen C, Nieder C, Vonen B. Contribution of adverse events to death of hospitalised patients. BMJ Open Qual. 2019;8(1):e000377. Epub 2019/04/19. doi: 10.1136/bmjoq-2018-000377.
22. Hoffmann-Volkl G, Kastenbauer T, Muck U, Zottl M, Huf W, Ettl B. [Detection of adverse events using IHI Global Trigger Tool during the adoption of a risk management system: A retrospective study over three years at a department for cardiovascular surgery in Vienna]. Z Evid Fortbild Qual Gesundhwes. 2018;131-132:38-45. Epub 2017/11/07. doi: 10.1016/j.zefq.2017.09.013.
23. Hommel A, Magneli M, Samuelsson B, Schildmeijer K, Sjostrand D, Goransson KE, et al. Exploring the incidence and nature of nursing-sensitive orthopaedic adverse events: A multicenter cohort study using Global Trigger Tool. Int J Nurs Stud. 2020;102:103473. Epub 2019/12/07. doi: 10.1016/j.ijnurstu.2019.103473.
24. Magneli M, Unbeck M, Rogmark C, Rolfson O, Hommel A, Samuelsson B, et al. Validation of adverse events after hip arthroplasty: a Swedish multi-centre cohort study. BMJ Open. 2019;9(3):e023773. Epub 2019/03/10. doi: 10.1136/bmjopen-2018-023773.
25. Magneli M, Unbeck M, Samuelsson B, Rogmark C, Rolfson O, Gordon M, et al. Only 8% of major preventable adverse events after hip arthroplasty are filed as claims: a Swedish multi-center cohort study on 1,998 patients. Acta Orthop. 2020;91(1):20-5. Epub 2019/10/17. doi: 10.1080/17453674.2019.1677382.
26. Hu Q, Wu B, Zhan M, Jia W, Huang Y, Xu T. Adverse events identified by the global trigger tool at a university hospital: A retrospective medical record review. J Evid Based Med. 2019;12(2):91-7. Epub 2018/12/05. doi: 10.1111/jebm.12329.
27. Hwang JI, Chin HJ, Chang YS. Characteristics associated with the occurrence of adverse events: a retrospective medical record review using the Global Trigger Tool in a fully digitalized tertiary teaching hospital in Korea. J Eval Clin Pract. 2014;20(1):27-35. Epub 2013/07/31. doi: 10.1111/jep.12075.
28. Kaibel Val R, Ruiz López P, Pérez Zapata AI, Gómez de la Cámara A, de la Cruz Vigo F. [Detection of adverse events in thyroid and parathyroid surgery using trigger tool and Minimum Basic Data Set (MBDS)]. J Healthc Qual Res. 2020;35(6):348-54. Epub 2020/10/30. doi: 10.1016/j.jhqr.2020.08.001.
29. Kelly-Pettersson P, Sköldenberg O, Samuelsson B, Stark A, Muren O, Unbeck M. The identification of adverse events in hip fracture patients using the Global Trigger Tool: A prospective observational cohort study. Int J Orthop Trauma Nurs. 2020;38:100779. Epub 2020/05/23. doi: 10.1016/j.ijotn.2020.100779.
30. Good VS, Saldana M, Gilder R, Nicewander D, Kennerly DA. Large-scale deployment of the Global Trigger Tool across a large hospital system: refinements for the characterisation of adverse events to support patient safety learning opportunities. BMJ Qual Saf. 2011;20(1):25-30. Epub 2011/01/14. doi: 10.1136/bmjqs.2008.029181.
31. Kennerly DA, Kudyakov R, da Graca B, Saldana M, Compton J, Nicewander D, et al. Characterization of adverse events detected in a large health care delivery system using an enhanced global trigger tool over a five-year interval. Health Serv Res. 2014;49(5):1407-25. Epub 2014/03/19. doi: 10.1111/1475-6773.12163.
32. Kennerly DA, Saldana M, Kudyakov R, da Graca B, Nicewander D, Compton J. Description and evaluation of adaptations to the global trigger tool to enhance value to adverse event reduction efforts. J Patient Saf. 2013;9(2):87-95. Epub 2013/01/22. doi: 10.1097/PTS.0b013e31827cdc3b.

33. Kurutkan MN, Usta E, Orhan F, Simsekler MC. Application of the IHI Global Trigger Tool in measuring the adverse event rate in a Turkish healthcare setting. Int J Risk Saf Med. 2015;27(1):11-21. Epub 2015/03/15. doi: 10.3233/JRS-150639.
34. Landrigan CP, Parry GJ, Bones CB, Hackbarth AD, Goldmann DA, Sharek PJ. Temporal trends in rates of patient harm resulting from medical care. N Engl J Med. 2010;363(22):2124-34. Epub 2010/11/26. doi: 10.1056/NEJMsa1004404.
35. Sharek PJ, Parry G, Goldmann D, Bones K, Hackbarth A, Resar R, et al. Performance characteristics of a methodology to quantify adverse events over time in hospitalized patients. Health Serv Res. 2011;46(2):654-78. Epub 2010/08/21. doi: 10.1111/j.1475-6773.2010.01156.x.
36. Lipczak H, Knudsen JL, Nissen A. Safety hazards in cancer care: findings using three different methods. BMJ Qual Saf. 2011;20(12):1052-6. Epub 2011/06/30. doi: 10.1136/bmjqs.2010.050856.
37. Lipczak H, Neckelmann K, Steding-Jessen M, Jakobsen E, Knudsen JL. Uncertain added value of Global Trigger Tool for monitoring of patient safety in cancer care. Dan Med Bull. 2011;58(11):A4337. Epub 2011/11/04.
38. Lipitz-Snyderman A, Classen D, Pfister D, Killen A, Atoria CL, Fortier E, et al. Performance of a Trigger Tool for Identifying Adverse Events in Oncology. J Oncol Pract. 2017;13(3):e223-e30. Epub 2017/01/18. doi: 10.1200/JOP.2016.016634.
39. Mattsson TO, Knudsen JL, Lauritsen J, Brixen K, Herrstedt J. Assessment of the global trigger tool to measure, monitor and evaluate patient safety in cancer patients: reliability concerns are raised. BMJ Qual Saf. 2013;22(7):571-9. Epub 2013/03/01. doi: 10.1136/bmjqs-2012-001219.
40. Mattsson TO, Knudsen JL, Brixen K, Herrstedt J. Does adding an appended oncology module to the Global Trigger Tool increase its value? Int J Qual Health Care. 2014;26(5):553-60. Epub 2014/08/01. doi: 10.1093/intqhc/mzu072.
41. Mayor S, Baines E, Vincent C, Lankshear A, Edwards A, Aylward M, et al. Measuring harm and informing quality improvement in the Welsh NHS: the longitudinal Welsh national adverse events study. Health Serv Deliv Res. 2017;5(9). doi: 10.3310/hsdr05090.
42. Menéndez-Fraga MD, Alonso J, Cimadevilla B, Cueto B, Vazquez F. Does Skilled Nursing Facility Trigger Tool used with Global Trigger Tool increase its value for adverse events evaluation? J Healthc Qual Res. 2021;36(2):75-80. Epub 2021/01/30. doi: 10.1016/j.jhqr.2020.08.004.
43. Mevik K, Griffin FA, Hansen TE, Deilkas ET, Vonen B. Does increasing the size of bi-weekly samples of records influence results when using the Global Trigger Tool? An observational study of retrospective record reviews of two different sample sizes. BMJ Open. 2016;6(4):e010700. Epub 2016/04/27. doi: 10.1136/bmjopen-2015-010700.
44. Mevik K, Griffin FA, Hansen TE, Deilkas ET, Vonen B. Is inter-rater reliability of Global Trigger Tool results altered when members of the review team are replaced? Int J Qual Health Care. 2016;28(4):492-6. Epub 2016/06/11. doi: 10.1093/intqhc/mzw054.
45. Moraes SM, Ferrari TCA, Figueiredo NMP, Almeida TNC, Sampaio CCL, Andrade YCP, et al. Assessment of the reliability of the IHI Global Trigger Tool: new perspectives from a Brazilian study. Int J Qual Health Care. 2021;33(1). Epub 2021/03/07. doi: 10.1093/intqhc/mzab039.
46. Mortaro A, Moretti F, Pascu D, Tessari L, Tardivo S, Pancheri S, et al. Adverse Events Detection Through Global Trigger Tool Methodology: Results From a 5-Year Study in an Italian Hospital and Opportunities to Improve Interrater Reliability. J Patient Saf. 2017. Epub 2017/06/10. doi: 10.1097/PTS.0000000000000381.
47. Mull HJ, Brennan CW, Folkes T, Hermos J, Chan J, Rosen AK, et al. Identifying Previously Undetected Harm: Piloting the Institute for Healthcare Improvement's Global Trigger Tool in the Veterans Health Administration. Qual Manag Health Care. 2015;24(3):140-6. Epub 2015/06/27. doi: 10.1097/QMH.0000000000000060.
48. Müller MM, Gous AG, Schellack N. Measuring adverse events using a trigger tool in a paper based patient information system at a teaching hospital in South Africa. Eur J Clin Pharm. 2016;18(2):103-12.

49. Naessens JM, Campbell CR, Huddleston JM, Berg BP, Lefante JJ, Williams AR, et al. A comparison of hospital adverse events identified by three widely used detection methods. Int J Qual Health Care. 2009;21(4):301-7. Epub 2009/07/21. doi: 10.1093/intqhc/mzp027.
50. Naessens JM, O'Byrne TJ, Johnson MG, Vansuch MB, McGlone CM, Huddleston JM. Measuring hospital adverse events: assessing inter-rater reliability and trigger performance of the Global Trigger Tool. Int J Qual Health Care. 2010;22(4):266-74. Epub 2010/06/11. doi: 10.1093/intqhc/mzq026.
51. Najjar S, Hamdan M, Euwema MC, Vleugels A, Sermeus W, Massoud R, et al. The Global Trigger Tool shows that one out of seven patients suffers harm in Palestinian hospitals: challenges for launching a strategic safety plan. Int J Qual Health Care. 2013;25(6):640-7. Epub 2013/10/22. doi: 10.1093/intqhc/mzt066.
52. Nilsson L, Risberg MB, Montgomery A, Sjodahl R, Schildmeijer K, Rutberg H. Preventable Adverse Events in Surgical Care in Sweden: A Nationwide Review of Patient Notes. Medicine (Baltimore). 2016;95(11):e3047. Epub 2016/03/18. doi: 10.1097/MD.0000000000003047.
53. Gunningberg L, Sving E, Hommel A, Alenius C, Wiger P, Baath C. Tracking pressure injuries as adverse events: National use of the Global Trigger Tool over a 4-year period. J Eval Clin Pract. 2019;25(1):21-7. Epub 2018/07/22. doi: 10.1111/jep.12996.
54. Nilsson L, Borgstedt-Risberg M, Soop M, Nylen U, Alenius C, Rutberg H. Incidence of adverse events in Sweden during 2013-2016: a cohort study describing the implementation of a national trigger tool. BMJ Open. 2018;8(3):e020833. Epub 2018/04/01. doi: 10.1136/bmjopen-2017-020833.
55. Nowak B, Schwendimann R, Lyrer P, Bonati LH, De Marchis GM, Peters N, et al. Occurrence of No-Harm Incidents and Adverse Events in Hospitalized Patients with Ischemic Stroke or TIA: A Cohort Study Using Trigger Tool Methodology. Int J Environ Res Public Health. 2022;19(5). Epub 2022/03/11. doi: 10.3390/ijerph19052796.
56. O'Leary KJ, Devisetty VK, Patel AR, Malkenson D, Sama P, Thompson WK, et al. Comparison of traditional trigger tool to data warehouse based screening for identifying hospital adverse events. BMJ Qual Saf. 2013;22(2):130-8. Epub 2012/10/06. doi: 10.1136/bmjqs-2012-001102.
57. Perez Zapata AI, Gutierrez Samaniego M, Rodriguez Cuellar E, Gomez de la Camara A, Ruiz Lopez P. [Comparison of the "Trigger" tool with the minimum basic data set for detecting adverse events in general surgery]. Rev Calid Asist. 2017;32(4):209-14. Epub 2017/03/21. doi: 10.1016/j.cali.2017.01.001.
58. Pérez Zapata AI, Gutiérrez Samaniego M, Rodríguez Cuéllar E, Andrés Esteban EM, Gómez de la Cámara A, Ruiz López P. Detection of Adverse Events in General Surgery Using the “Trigger Tool” Methodology. Cirugía Española (English Edition). 2015;93(2):84-90. doi: 10.1016/j.cireng.2014.12.005.
59. Pérez Zapata AI, Rodríguez Cuéllar E, de la Fuente Bartolomé M, Martín-Arriscado Arroba C, García Morales MT, Loinaz Segurola C, et al. Predictive Power of the "Trigger Tool" for the detection of adverse events in general surgery: a multicenter observational validation study. Patient Saf Surg. 2022;16(1):7. Epub 2022/02/10. doi: 10.1186/s13037-021-00316-3.
60. Pierdevara L, Porcel-Gálvez AM, Ferreira da Silva AM, Barrientos Trigo S, Eiras M. Translation, Cross-Cultural Adaptation, and Measurement Properties of the Portuguese Version of the Global Trigger Tool for Adverse Events. Ther Clin Risk Manag. 2020;16:1175-83. Epub 2020/12/11. doi: 10.2147/tcrm.S282294.
61. Rutberg H, Borgstedt Risberg M, Sjodahl R, Nordqvist P, Valter L, Nilsson L. Characterisations of adverse events detected in a university hospital: a 4-year study using the Global Trigger Tool method. BMJ Open. 2014;4(5):e004879. Epub 2014/05/30. doi: 10.1136/bmjopen-2014-004879.
62. Rutberg H, Borgstedt-Risberg M, Gustafson P, Unbeck M. Adverse events in orthopedic care identified via the Global Trigger Tool in Sweden - implications on preventable prolonged hospitalizations. Patient Saf Surg. 2016;10:23. Epub 2016/11/02. doi: 10.1186/s13037-016-0112-y.

63. Schildmeijer K, Nilsson L, Arestedt K, Perk J. Assessment of adverse events in medical care: lack of consistency between experienced teams using the global trigger tool. BMJ Qual Saf. 2012;21(4):307-14. Epub 2012/03/01. doi: 10.1136/bmjqs-2011-000279.
64. Sekijima A, Sunga C, Bann M. Adverse events experienced by patients hospitalized without definite medical acuity: A retrospective cohort study. J Gen Intern Med. 2020;34(2):S125. doi: 10.1007/11606.1525-1497.
65. Suarez C, Menendez MD, Alonso J, Castano N, Alonso M, Vazquez F. Detection of adverse events in an acute geriatric hospital over a 6-year period using the Global Trigger Tool. J Am Geriatr Soc. 2014;62(5):896-900. Epub 2014/04/05. doi: 10.1111/jgs.12774.
66. Menendez Fraga MD, Cueva Alvarez MA, Franco Castellanos MR, Fernandez Moral V, Castro Del Rio MP, Arias Perez JI, et al. [Compliance with the surgical safety checklist and surgical events detected by the Global Trigger Tool]. Rev Calid Asist. 2016;31 Suppl 1:20-3. Epub 2016/06/07. doi: 10.1016/j.cali.2016.03.006.
67. Toribio-Vicente MJ, Chalco-Orrego JP, Diaz-Redondo A, Llorente-Parrado C, Pla-Mestre R. [Detection of adverse events using trigger tools in 2hospital units in Spain]. J Healthc Qual Res. 2018;33(4):199-205. Epub 2018/01/01. doi: 10.1016/j.jhqr.2018.05.003.
68. Unbeck M, Schildmeijer K, Henriksson P, Jurgensen U, Muren O, Nilsson L, et al. Is detection of adverse events affected by record review methodology? an evaluation of the "Harvard Medical Practice Study" method and the "Global Trigger Tool". Patient Saf Surg. 2013;7(1):10. Epub 2013/04/17. doi: 10.1186/1754-9493-7-10.
69. von Plessen C, Kodal AM, Anhoj J. Experiences with global trigger tool reviews in five Danish hospitals: an implementation study. BMJ Open. 2012;2(5). Epub 2012/10/16. doi: 10.1136/bmjopen-2012-001324.
70. Wilson RM, Michel P, Olsen S, Gibberd RW, Vincent C, El-Assady R, et al. Patient safety in developing countries: retrospective estimation of scale and nature of harm to patients in hospital. BMJ. 2012;344:e832. Epub 2012/03/15. doi: 10.1136/bmj.e832.
71. Xu XD, Yuan YJ, Zhao LM, Li Y, Zhang HZ, Wu H. Adverse Events at Baseline in a Chinese General Hospital: A Pilot Study of the Global Trigger Tool. J Patient Saf. 2020;16(4):269-73. Epub 2016/09/10. doi: 10.1097/PTS.0000000000000329.
72. Zadvinskis IM, Salsberry PJ, Chipps EM, Patterson ES, Szalacha LA, Crea KA. An Exploration of Contributing Factors to Patient Safety. J Nurs Care Qual. 2018;33(2):108-15. Epub 2018/02/22. doi: 10.1097/NCQ.0000000000000284.
