# Supplementary figures and images for: Variation in detected adverse events using trigger tools: A systematic review and meta-analysis
Source: PLoS One. 2022 Sep 1;17(9):e0273800. doi: 10.1371/journal.pone.0273800 (PMC9436152; doi:10.1371/journal.pone.0273800)

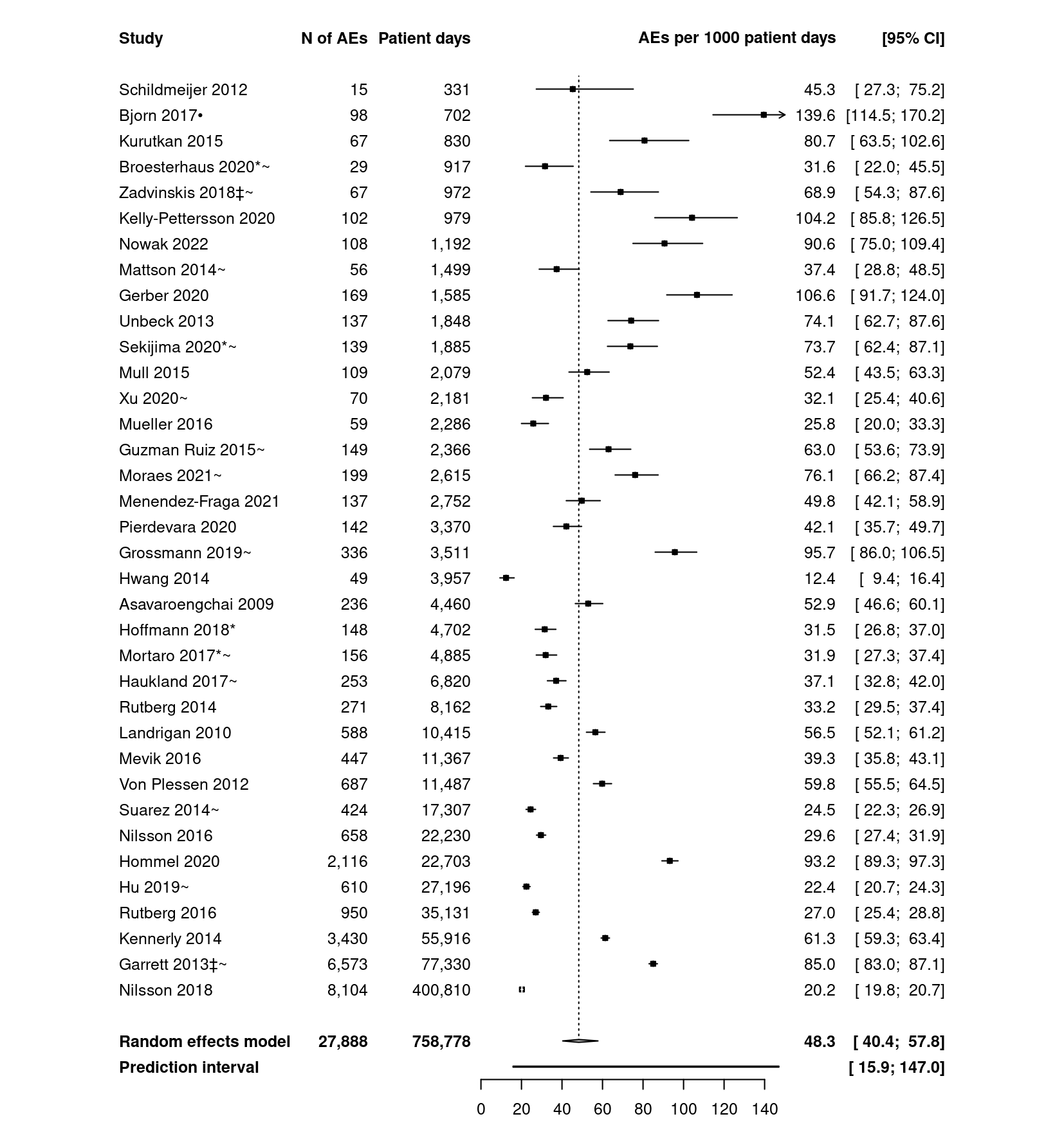

Supplement: S1 Fig — * = pooled estimate, • = mean estimate, ‡ = calculated total number of AEs, ~ = calculated total number of patient days [5, 15, 17–22, 34, 37, 39, 40, 45, 46, 50–52, 54, 57, 58, 60, 62–65, 67, 68, 72, 73, 76–79, 82, 84–87, 89–91, 93, 95, 96, 98–100, 102]. (TIF) [file pone.0273800.s004.tif]

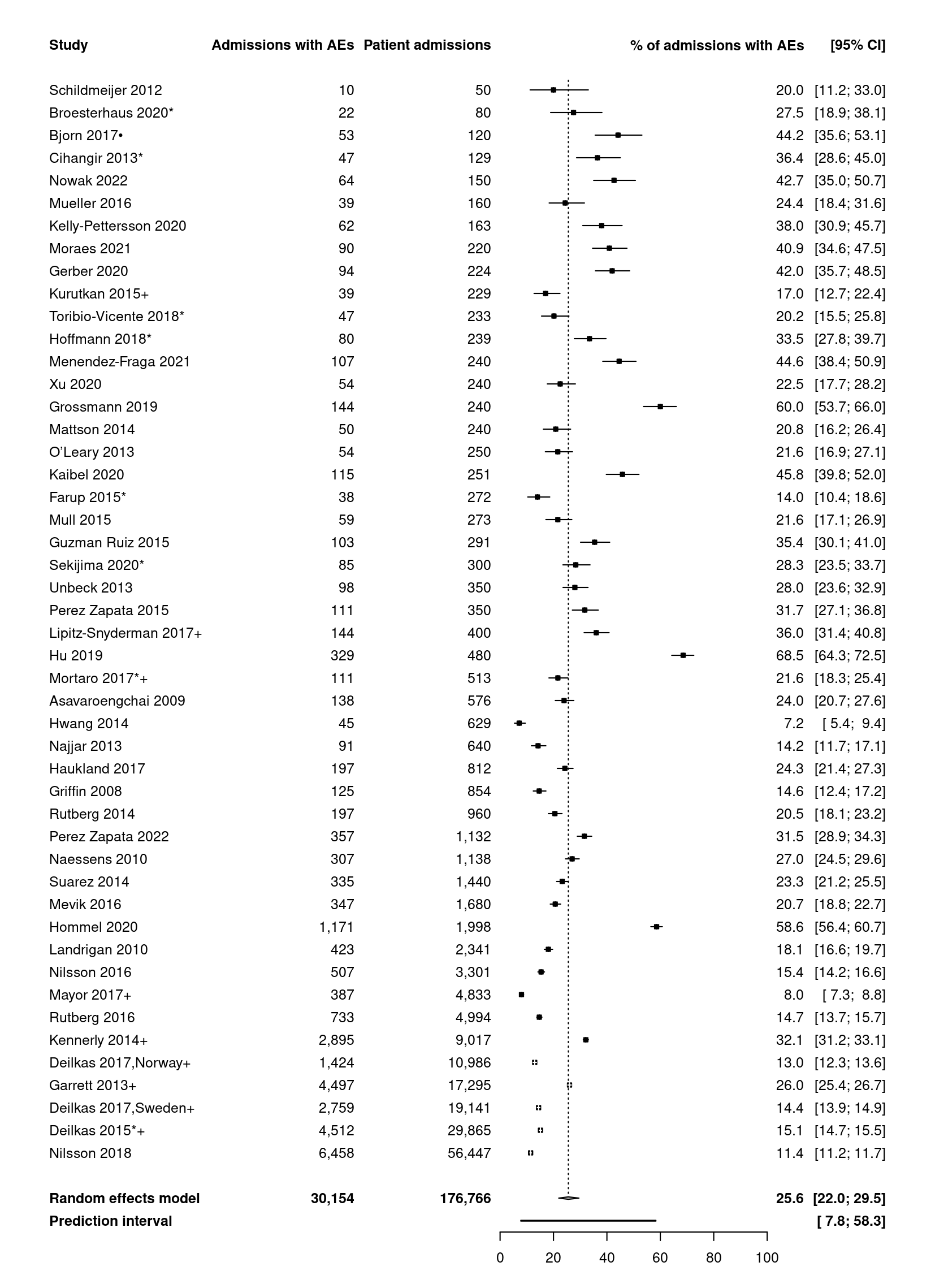

Supplement: S2 Fig — CI, confidence interval; * = pooled estimate, • = mean estimate, + = calculated total number of admissions with ≥ 1 AE [5, 9, 14, 15, 17–22, 24, 34, 37, 39, 45, 46, 50–58, 60–68, 70, 72–87, 89–94, 96–101]. (TIF) [file pone.0273800.s005.tif]

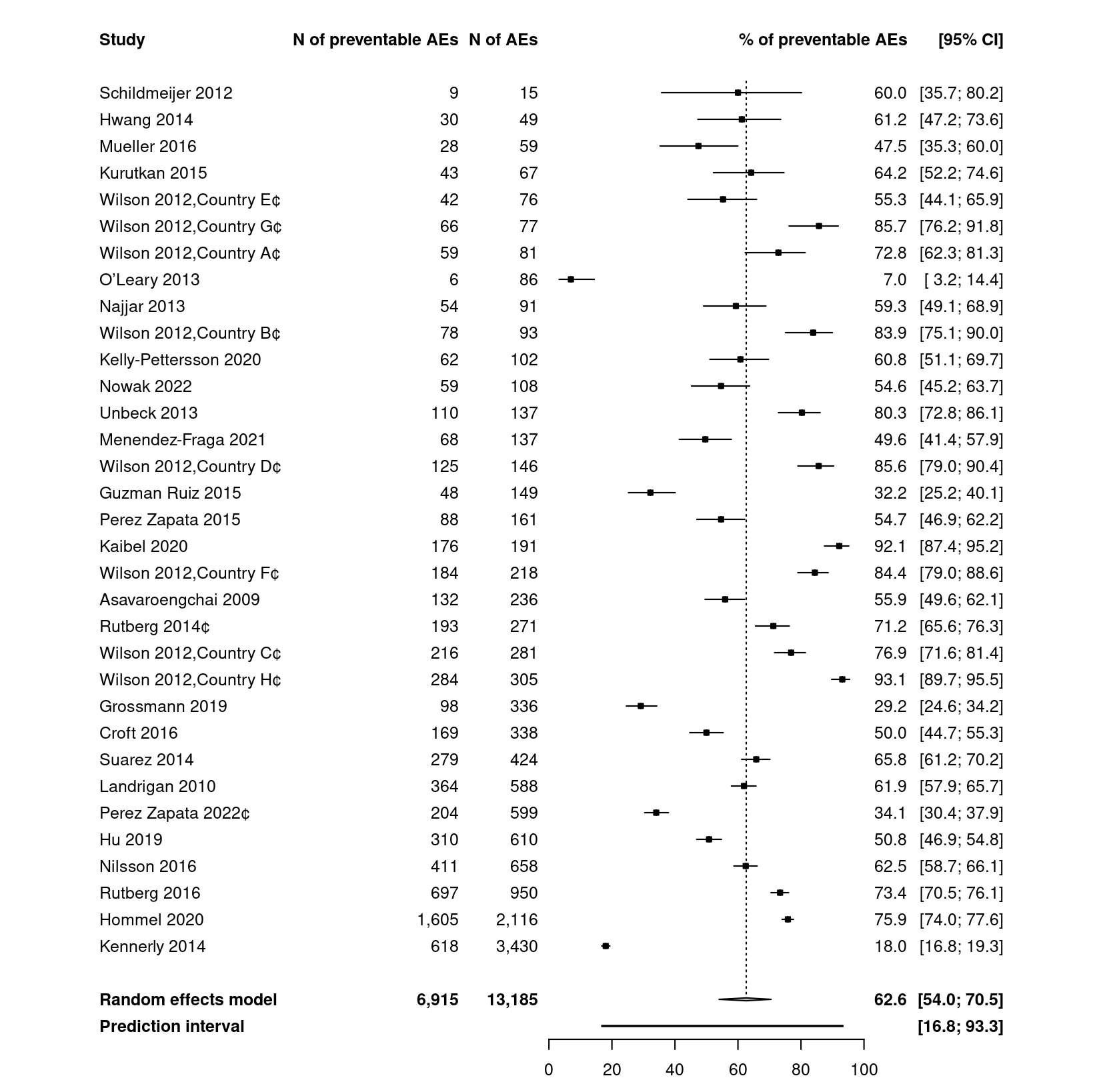

Supplement: S3 Fig — CI, confidence interval; * = pooled estimate, • = mean estimate, ¢ = calculated number of preventable AEs [15, 17–20, 34, 37–39, 46, 50, 51, 53, 59, 63–67, 71–75, 77, 78, 87, 89–91, 96–98, 100, 101]. (TIF) [file pone.0273800.s006.tif]

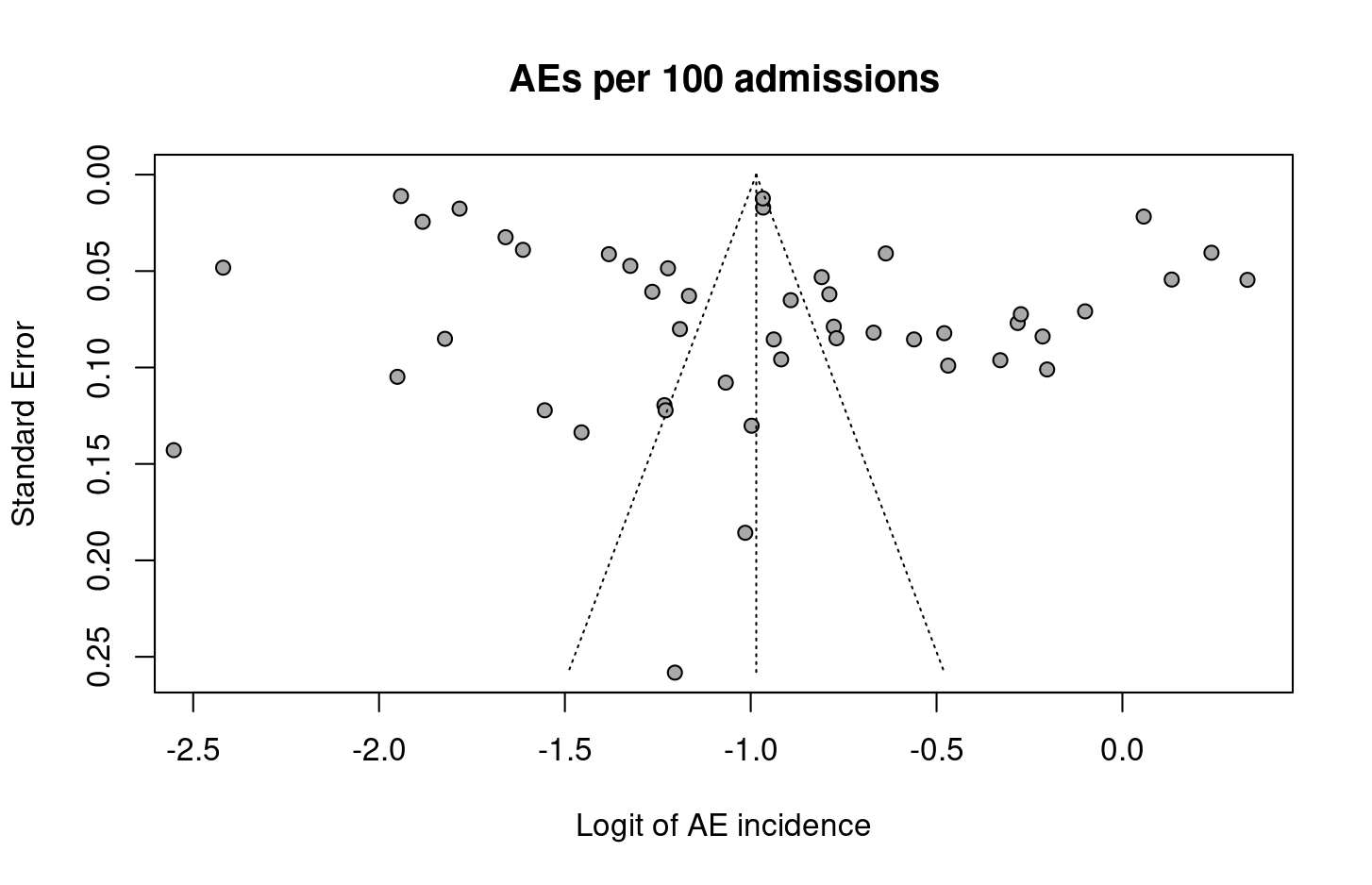

Supplement: S4 Fig — (TIF) [file pone.0273800.s007.tif]

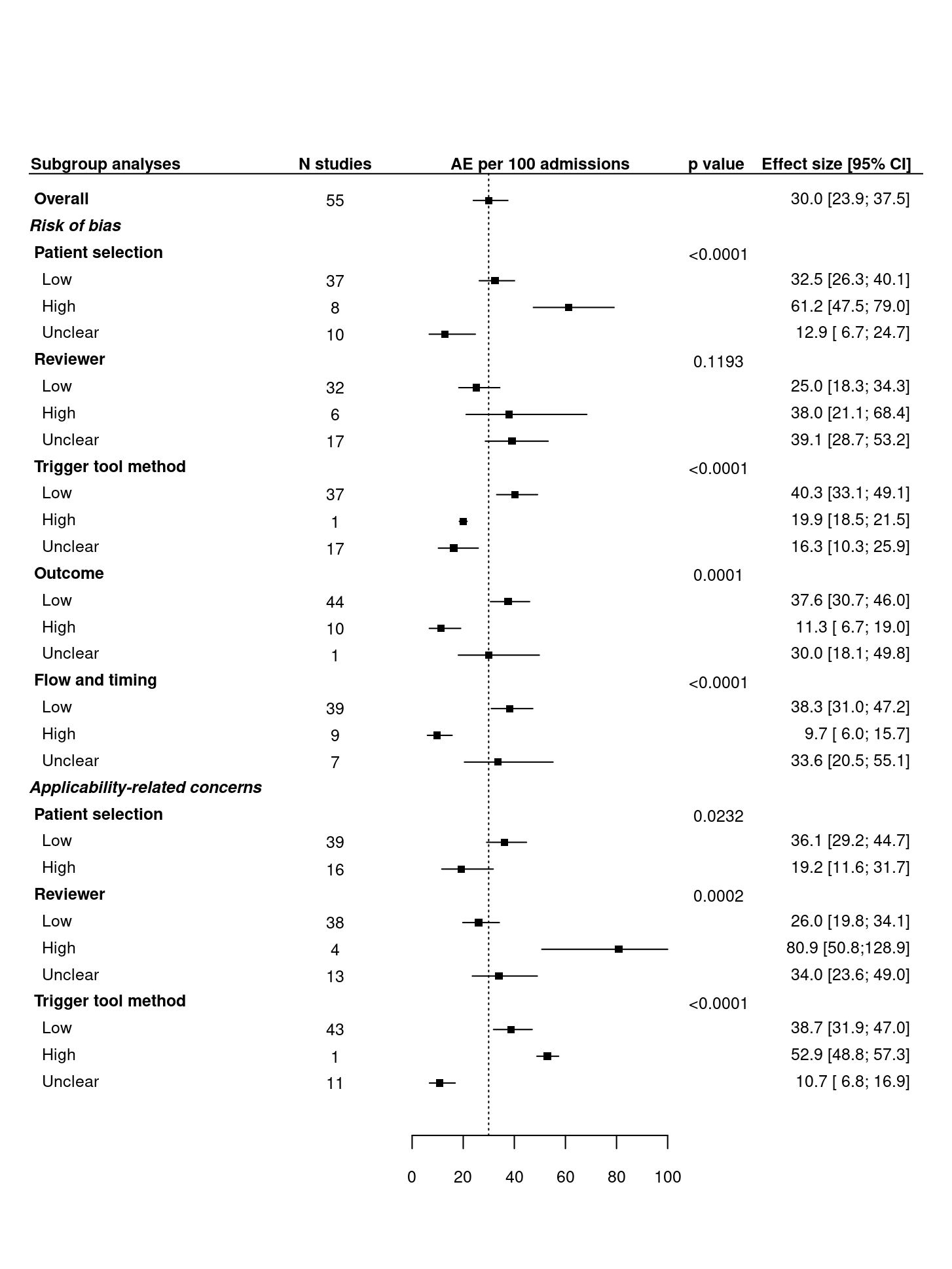

Supplement: S5 Fig — AE, adverse events; N studies, number of studies; CI, confidence interval [5, 10, 15, 17–22, 34, 37–39, 45, 46, 50–54, 56–69, 71–79, 82–91, 93, 95–102]. (TIF) [file pone.0273800.s008.tif]
