# Supplementary material for: Variation in detected adverse events using trigger tools: A systematic review and meta-analysis
Source: PLoS One. 2022 Sep 1;17(9):e0273800. doi: 10.1371/journal.pone.0273800 (PMC9436152; doi:10.1371/journal.pone.0273800)
Supplement: Attachment — Submitted filename: PONE-D-21-40420-adverse events SR-reviewed.pdf [file pone.0273800.s009.pdf]

TITLE

The title is clear with enough detail for the reader to know what to expect.

RELEVANCE AND ORIGINALITY

Adverse events are an ongoing occurrence in the health landscape and the mechanism of identifying and reporting adverse events is not consistent across or between countries. This review is relevant as it provides an argument (using a recognised high quality and rigorous approach, i.e., a systematic review and meta-analysis) for the need to address this inconsistency with clearer reporting guidance.

AUTHENTICITY AND REFERENCING

The manuscript appears to be the work of the author with appropriate attribution to the work of others both in text and in the reference list.

ABSTRACT/INTRODUCTION

The abstract is comprehensive and an accurate reflection of the manuscript. The introduction is brief yet provides enough information from the literature to support the need for the review. In addition, key terms, (e.g., 'global trigger tool', 'trigger') are explained and operationalised for the review. The introduction leads logically to the gap in the literature and the aims of the study.

AIMS

Dual aims are clear.

METHODOLOGY

The methodology is well described and replicable, apart from a few queries:

- One evidence source searched, 'all authors' personal libraries' (line 117), is not defined or described. Are the authors referring to self -authored publications or simply publications amassed in personal collections? If the former, then these papers would presumably be indexed in one of the other databases searched. If the latter, then it renders the search not replicable. Removal of 'personal libraries' or explanation for its inclusion might address any concerns raised by its inclusion.
- Similarly, an explanation for the choice of the three journals hand searched would allay any concerns of bias in the search strategy.

Re eligibility criteria – point #3 is "…acute care (including elective admissions) hospital settings… (line 122). I would think elective admissions are inherently a part of an acute care cohort. Did the authors mean 'emergency admissions'? In either case, this could be clarified.

Table 1 is particularly helpful.

RESULTS

Results are comprehensive and well organised.

TABLES AND FIGURES

Tables and figures are well presented and do not replicate information in text.

DISCUSSION/CONCLUSION

The discussion is supported by the findings and the findings are situated within the current body of evidence on the topic. Recommendations for future practice related to adverse events and future research reporting on adverse events, albeit very brief (e.g., one sentence each), logically derives from the findings and discussion.

OTHER COMMENTS

Use of a reporting guideline is not evident but is a conventional expectation. The authors might consider adding reference to this in some way.

WRITING STYLE

The writing style is easy to academically sound and easy to read.

SCHOLARLY APPROACH

The authors have used a scholarly approach that begins with a clearly stated premise so that compelling arguments can be presented and supported with up-to-date literature, including empirical research evidence. Providing more critique of the studies cited in the introduction and in the discussion would elevate this further.

OVERALL COMMENTS

My comments have been provided in the spirit of collegiality to hopefully assist the authors in further preparing their manuscript for publication. I commend the authors on this high-quality report of their systematic review and meta-analysis.
