# Supplementary material for: Variation in detected adverse events using trigger tools: A systematic review and meta-analysis
Source: PLoS One. 2022 Sep 1;17(9):e0273800. doi: 10.1371/journal.pone.0273800 (PMC9436152; doi:10.1371/journal.pone.0273800)
Supplement: Attachment — Submitted filename: Response to Reviewers.docx [file pone.0273800.s010.docx]

To the Editor:

We appreciate the opportunity to address the reviewers’ comments and revise our manuscript. Below, please find item-by-item responses to the Reviewers’ comments, which are included verbatim. All page and paragraph numbers refer to locations in the revised manuscript.

**REVIEWER 1 COMMENTS – MAJOR**

COMMENT 1:

The overall message of the study is difficult to follow, you report many results and subgroups(?) and these are not specified in the method section. Could you rearrange the result section with subheadings or omit some of the analyses to guide the reader.

RESPONSE 1:

We gratefully thank the reviewer for the remark. We made adaptations to the results section to increase the flow and clarity of the main results.

COMMENT 2:

The date of search is difficult to find, and it seems that the date of the last search >1,5-2 years ago. The search should be updated.

RESPONSE 2:

We conducted an update search 25. Mai 2022. We add the date of the search in the abstract as well as the method section. The full manuscript includes now six new studies which were published between 2020 and 2022.

We have amended the manuscript throughout.

COMMENT 3:

You state in the method section that PRISMA 2020 was identified but several items and the flow diagram from PRISMA 2020 is missing. I have listed some below in minor revisions but I recommend that you upload a PRISMA 2020 checklist stating where each item can be located.

RESPONSE 3:

We agree that not all PRISMA 2020 items were properly addressed. We adapted the flow diagram according to the PRISMA guidelines example and will point out where we have made changes in the following section.

**REVIEWER 1 COMMENTS – MINOR**

COMMENT 1:

Acute care or acute-care? Please uniform

RESPONSE 1:

We have changed the notation to acute care throughout the manuscript (Line 29, Page 02).

COMMENT 2

Incidence or prevalence

RESPONSE 2

We are reporting solely incidences and have changed this throughout the manuscript (Table 1, Page 08).

COMMENT 3

Abstract: please add the eligibility criteria on language and exclusion criteria that you describe in the method section.

RESPONSE 3

Thanks for indicating the missing information of inclusion and exclusion criteria in the abstract, which we have added (Lines 37-38, Page 02).

COMMENT 4

Abstract: Please provide the date last searched (PRISMA 2020 for abstracts checklist. Item 4: https://www.equator-network.org/reporting-guidelines/prisma-abstracts/)

RESPONSE 4

We have added the exact date now in the methods section in the abstract (Line 34, Page 02).

COMMENT 5

Abstract: Please describe methods to assess risk of bias (PRISMA 2020 for abstracts checklist. Item 5: https://www.equator-network.org/reporting-guidelines/prisma-abstracts/)

RESPONSE 5

We have added a sentence about the tool (Lines 38-40, Page 02).

COMMENT 6

Abstract: Please report I^2

RESPONSE 6

We have added the I^2 of 99.7% for the main outcome (Line 44, Page 02).

COMMENT 7

Abstract: I do not understand the results, could you simply? Several terms have not been introduced: e.g. applicability-related concerns, commission and omission, reviewers’ level of experience, the evidence on the remainder.

RESPONSE 7

Thank you for the comment. We have simplified the results section and focused on key results (Lines 43-48, Page 02).

COMMENT 8

Abstract: Please provide details on registration and funding (PRISMA 2020 for abstracts checklist. Item 11+12: https://www.equator-network.org/reporting-guidelines/prisma-abstracts/)

RESPONSE 8

We have added this information at the end of the abstract’s method section (Line 42, Page 02). We did not include this information before, as we did not receive any funding and we did not register the review protocol beforehand. Although we tried to adhere to the 300 words limit in the abstract we will now submit a version that will exceed the 300 words limitation. It seems impossible to make the 300-word limit and including all PRISMA 2020 items.

COMMENT 9

Your REF 2 is the Trigger Tool – would ICH GCP not be better suited?

RESPONSE 9

In this review, we have identified several ways of defining adverse events, including the definition of the ICH GCP. As we have only included studies that applied the Global Trigger Tool (GTT) and its variations we refer to the IHI White Paper for the adverse event definition which is the basis for the GTT. This seems the best match between the background and the applied methodologies. Therefore, we suggest to keep REF 2.

COMMENT 10

Consider using the term from the cited reference [8] “medical record review” rather that “record review” throughout your article.

RESPONSE 10

We have adapted it throughout the manuscript.

COMMENT 11

Introduction: Please revise sentence and commas for: “Record review uses available data [8], was found to identify more AEs when 70 compared with many other methods [9, 10], can be repeated over time and can target specific AE 71 types, or the overall AE rate [11].”

RESPONSE 11

Thanks for pointing out the unclear sentence. We revised the sentence and hope it is clearer now (Lines 66-68, Page 04).

COMMENT 12

Introduction: Please correct: “A "trigger" (or clue) consists either of specific wording or an event in a medical 82 record that could indicate the occurrence of an AE, e.g., readmissions within 30 days or pressure 83 ulcers [2].”

RESPONSE 12

We have revised the sentence to make it easier to read (Lines 78-80, Page 05).

COMMENT 13

Methods: Please revise: “Design Systematic review and meta-analyses [27].” So that it reflect that you reported according to PRISMA 2020 [27].

RESPONSE 13

We agree that it was not clearly stated that we adhere to PRISMA 2020. We have now revised the sentence to clarify this (Lines 105-106, Page 06).

COMMENT 14

Methods: Should the subheading “data sources” rather be “information sources” (PRISMA 2020, item 6)?

RESPONSE 14

We have changed the subheading to “search strategy and information sources” (Line 107, Page 06).

COMMENT 15

Methods: Your specific search strategy is difficult to follow:

a. You write that “The medical subject headings (MeSH) and keywords for titles and abstracts” was your search limited to title and abstract or were all fields searched (PRISMA 2020, item 7)?

b. Was “medical error” combined with AND or put in quotation marks?

c. Which of your search terms were Mesh terms? How were these translated from PubMed to the other databases?

d. Please provide the full (and specific) search strategy to each database as recommended by PRISMA 2020, item 7.

e. You first state that “Our search strategy was developed and validated using methods suggested by Hausner et 111 al. [28, 29]. This involves generating a test set, developing and validating a search strategy and 112 documenting the strategy using a standardized approach [29]” but later that “The detailed search strategy used 119 for this review is that described by Musy et al. [26].” – did you or reference [26] develop the search strategy and applied the methods.

RESPONSE 15

Thank you for the remark. We have added now additional information about the strategy with information on title and abstracts as well as mesh terms. The search string shown now was used exactly as written. “Medical error” was used as a MeSH term. The author SM did apply the identical search, as was done in the reference [26]. The strategy is published in Musy et al. 2018 (Musy SN, Ausserhofer D, Schwendimann R, Rothen HU, Jeitziner MM, Rutjes AW, et al. Trigger Tool-Based Automated Adverse Event Detection in Electronic Health Records: Systematic Review. J Med Internet Res. 2018;20(5):e198. Epub 2018/06/01. doi: 10.2196/jmir.9901) (Lines 111-118, Page 06).

COMMENT 16

Methods: Please provide date of last search (PRISMA 2020, 6), if the date of last search is >12 months ago I recommend that you update the search.

RESPONSE 16

We have moved the information on the index search (Nov 20015) and the following update searches (Apr 2016, Jul 2017, Jan 2020, Sep 2020 and 25. Mai 2022) from the results part to the method section and added additional data (months) and the specific date of the last search (Lines 118-120, Page 06).

COMMENT 17

Methods: from you flow diagram, it seems that you have a restriction on the search date (2015 and onwards”, please report this, PRISMA 2020, item 6.

RESPONSE 17

The flow diagram as it was, was misleading. As the dates were meant to be the index search (2015) and the following update searches. According to your suggestion, we have now adapted the flow diagram (see RESPONSE 25).

To address your question on restriction, we state in the search strategy and information sources section that publication dates were unrestricted (Lines 117, Page 06).

COMMENT 18

Methods: Were title and abstracts screened by one researcher or two researcher independently? Please specify in the manuscript.

RESPONSE 18

The two researchers did the title and abstracts screening independently, as well as the full-text screening. We have adapted the sentence accordingly (Lines 129-132, Page 07).

COMMENT 19

Please add details on data collection process, PRISMA 2020, item 9.

RESPONSE 19

We have added that the data was collected into an online data collection instrument (Lines 133-135, Page 07).

COMMENT 20

Please add information on PRISMA 2020, item 10b.

RESPONSE 20

We have now added some examples for the extraction information. We will make the extracted data publicly available after publication (Line 133-135, Page 07).

COMMENT 21

Methods: why did you have to invent a new bias assessment tool?

RESPONSE 21

We decided to adapt an existing tool that is designed to assess the risk of bias for diagnostic accuracy studies (QUADAS II). The trigger tool methodology is very similar to a diagnostic test but differs as it is not for diagnostic tests of patients. To us this seemed to be the best and most accurate way for the assessment of risk of bias as well as applicability related concerns. The adapted tool was used before in one review for automated trigger tool-based AE detection studies (Musy SN, Ausserhofer D, Schwendimann R, Rothen HU, Jeitziner MM, Rutjes AW, et al. Trigger Tool-Based Automated Adverse Event Detection in Electronic Health Records: Systematic Review. J Med Internet Res. 2018;20(5):e198. Epub 2018/06/01. doi: 10.2196/jmir.9901.). We decided to use this tool and adapted it slightly according to the authors described limitations of the tool.

COMMENT 22

Methods: How was heterogenicity measured, which cut-offs did you use?

RESPONSE 22

We have added that we considered a p<0.05 as significant in our subgroup analyses. We have also addressed heterogeneity by investigating prediction intervals, which is in our case more informative than I^2 due to the high values of I^2. We have added a reference that explains this further (IntHout J, Ioannidis JP, Rovers MM, Goeman JJ. Plea for routinely presenting prediction intervals in meta-analysis. BMJ Open. 2016;6(7):e010247. Epub 2016/07/14. doi: 10.1136/bmjopen-2015-010247.) (Lines 187-189, Page 10/11).

COMMENT 23

Methods: your approach “Because R's 176 metaprop function does not accept proportions exceeding 100%, we adapted results of four 177 studies where the number of AEs exceeded the number of patient admissions. To reduce 178 oversized values to less than 100 AEs per 100 admissions, we reduced the number of AEs 179 detected to one less than the number of admissions (e.g., for a patient group of 240 with 336 180 AEs, we entered 239 AEs).” Can you provide a reference for this?

RESPONSE 23

We thank the reviewer for querying this. In the meantime we have found a different function in the meta package that allows us to use all data as reported. We adapted the methods part accordingly and deleted this section (Lines 176-180, Page 10).

COMMENT 24

Results: dates should be reported in methods.

RESPONSE 24

We moved the dates to the methods section (Lines 118-120, Page 06).

COMMENT 25

Results: Please help me understand your flow diagram – the layout of PRISMA 2020 has not been used. Did you use automatic tolls for the screening and exclusion of the 4531 non-trigger tools? Please specify in the method section if you did and only screened 406 title/abstracts independently. Only fulltext exclusion reasons must be explained in detail.

RESPONSE 25

We have now implemented the template from PRISMA 2020. We did not use any automatic tools for the first screening. We decided to do a first screening of title and abstracts with the sole inclusion criteria if it was anyhow trigger-tool related (two authors independently). In a second step, we again screened only title and abstracts for our formulated inclusion and exclusion criteria (two authors independently). In a third step, we then went to the full-text screenings. We added information in the methods part to explain this more clearly (Lines 129-132, Page 07 & Fig1. Flow diagram).

COMMENT 26

Results: Please correct: “The reviewed studies were all published between 2009 and 2020” to “included”.

RESPONSE 26

We have changed this accordingly (Line 203, Page 11).

COMMENT 27

Results: please uniform: “Overall, we included 192,316 index admissions in our report” in the abstract these as described as patients, which is more correct?

RESPONSE 27

We agree that this needs to be reported uniformly. We changed the abstract accordingly, as admissions is what we analysed (Line 43, Page 02).

COMMENT 28

Results: which type of studies was included in the review? Cohort studies, RCTs? This is not described in the method section or table 1.

RESPONSE 28

We have included all type of studies, except if they did not include outcome data relevant to our study. We have excluded other systematic reviews, which we now have added in the exclusion criteria (Line 126, Page 07).

COMMENT 29

Result: There are a lot of results reported in this section – and a lot of analysis. The section is difficult to follow and not all subgroups are evident from the method section. Could you omit so analysis or add some aiding subheadings?

RESPONSE 29

We have changed some subheadings and condensed the results somewhat. First, we describe an overview of the 54 included studies. Then we show how we have rated the risk of bias and concerns regarding applicability for those studies. Third, we display the results from the meta-analysis for our main outcome (AE per 100 admissions) and secondary outcomes (AE per 1,000 patient days, % of admissions with AEs and % of preventable AEs). Then we display the core outcome of our review. The explanation of heterogeneity of reported AE rates by eight out of nine study characteristics. In the end of the results part, we describe, how risk of bias and concerns regarding applicability influence the AE rates. (Full results section, especially lines 274-323, Pages 16-18).

COMMENT 30

Please add PRISMA 2020, item 22.

RESPONSE 30

We thank the reviewer for the comment. In the section “quality assessment” we display the figure with the risk of bias and applicability-related concerns and describe these results. As we don’t compare treatment effects, we have decided to not assess the studies’ quality along the GRADE assessment but applied the adapted version of the quality assessment instrument used for diagnostic test accuracy. Additionally, we applied a subgroup analysis on our main outcome with regard to the quality assessment. We now have added the individual results of the quality assessments per study in the appendix to make it more transparent how studies are rated (S1_Table) (Lines 247-248, Page 15).

COMMENT 31

Discussion: Please provide a key findings paragraph in the beginning of the discussion section with the key findings of your study without references to other studies.

RESPONSE 31

We have adapted the first section of the discussion and have added the key findings (Lines 328-336, Page 18/19).

COMMENT 32

Discussion: please expand your limitations section.

RESPONSE 32

We have supplemented the limitations section by several aspects, like the missing protocol registration and missing analysis on conflict of interests and funding. Furthermore, we have added a comment on the search strategy and non-screening of grey literature (Lines 400-421, Pages 22-23).

COMMENT 33

Did you analyse conflict of interest and funding of included studies and accounted for that in the analyses?

RESPONSE 33

The main outcome of our included studies are adverse event rates and we expected that this type of study is not influenced by financial drivers, as there are no comparative elements like in a medical treatment study. We have not included these elements in your meta-analysis, but it would be good for a future analysis to also investigate this topic.

**REVIEWER 2 COMMENTS**

COMMENT 1

TITLE

The title is clear with enough detail for the reader to know what to expect.

RELEVANCE AND ORIGINALITY

Adverse events are an ongoing occurrence in the health landscape and the mechanism of identifying and reporting adverse events is not consistent across or between countries. This review is relevant as it provides an argument (using a recognised high quality and rigorous approach, i.e., a systematic review and meta-analysis) for the need to address this inconsistency with clearer reporting guidance.

AUTHENTICITY AND REFERENCING

The manuscript appears to be the work of the author with appropriate attribution to the work of others both in text and in the reference list.

ABSTRACT/INTRODUCTION

The abstract is comprehensive and an accurate reflection of the manuscript. The introduction is brief yet provides enough information from the literature to support the need for the review. In addition, key terms, (e.g., ‘global trigger tool’, ‘trigger’) are explained and operationalised for the review. The introduction leads logically to the gap in the literature and the aims of the study.

AIMS

Dual aims are clear.

**RESPONSE 1**

We gratefully thank the reviewer for the insightful comments.

COMMENT 2

METHODOLOGY

The methodology is well described and replicable, apart from a few queries:

• One evidence source searched, ‘all authors’ personal libraries’ (line 117), is not defined or described.

Are the authors referring to self -authored publications or simply publications amassed in personal collections? If the former, then these papers would presumably be indexed in one of the other databases searched. If the latter, then it renders the search not replicable. Removal of ‘personal libraries’ or explanation for its inclusion might address any concerns raised by its inclusion.

**RESPONSE 2**

Indeed, we did include two publications that were only identifiable through personal libraries. We have now indicated the amount of studies identified through this source in the flow diagram. We also add this information in the limitations section, that those two studies were added (Lines 401-404, Page 22).

COMMENT 3

• Similarly, an explanation for the choice of the three journals hand searched would allay any concerns of bias in the search strategy.

**RESPONSE 3**

The research team identified the three journals as the ones most often publishing trigger tool adverse event studies. We have added a sentence that the search strategy and further information was published in Musy et al. 2018 (Musy SN, Ausserhofer D, Schwendimann R, Rothen HU, Jeitziner MM, Rutjes AW, et al. Trigger Tool-Based Automated Adverse Event Detection in Electronic Health Records: Systematic Review. J Med Internet Res. 2018;20(5):e198. Epub 2018/06/01. doi: 10.2196/jmir.9901.) (Lines 114-118, Page 06).

COMMENT 4

Re eligibility criteria – point #3 is “…acute care (including elective admissions) hospital settings… (line 122). I would think elective admissions are inherently a part of an acute care cohort. Did the authors mean ‘emergency admissions’? In either case, this could be clarified.

Table 1 is particularly helpful.

**RESPONSE 4**

“Elective admissions” refers to elective surgeries. We were not fully sure, if this is clear that elective admissions are inherently part of acute care. But since it seems to confuse the reader, we decided to remove this part (Line 123, Page 07).

COMMENT 5

RESULTS

Results are comprehensive and well organised.

TABLES AND FIGURES

Tables and figures are well presented and do not replicate information in text.

DISCUSSION/CONCLUSION

The discussion is supported by the findings and the findings are situated within the current body of evidence on the topic. Recommendations for future practice related to adverse events and future research reporting on adverse events, albeit very brief (e.g., one sentence each), logically derives from the findings and discussion.

**RESPONSE 5**

We gratefully thank the reviewer for the feedback.

COMMENT 6

OTHER COMMENTS

Use of a reporting guideline is not evident but is a conventional expectation. The authors might consider adding reference to this in some way.

**RESPONSE 6**

We have added the reference and information at the beginning of the results part and supplemented the online supplementary material with the PRISMA 2020 checklist (Lines 105-106, Page 06).

COMMENT 7

WRITING STYLE

The writing style is easy to academically sound and easy to read.

SCHOLARLY APPROACH

The authors have used a scholarly approach that begins with a clearly stated premise so that compelling arguments can be presented and supported with up-to-date literature, including empirical research evidence. Providing more critique of the studies cited in the introduction and in the discussion would elevate this further.

OVERALL COMMENTS

My comments have been provided in the spirit of collegiality to hopefully assist the authors in further preparing their manuscript for publication. I commend the authors on this high-quality report of their systematic review and meta-analysis.

**RESPONSE 7**

Thank you for your kind feedback. We agree that the discussion part would benefit from more critique of the studies cited in the introduction and discussion. Due to the short revision time frame, we have decided to focus on the update search, with the new results to be implemented.
